# Integrated Phytochemical Profiling, GC-MS Characterization, and In Silico, In Vitro Evaluation of Synergistic Antimicrobial, Antioxidant, and Anti-Inflammatory Activities of *Morus alba* Bark and *Pinus densiflora* Extracts with Methyl Gallate

**DOI:** 10.3390/antiox14091114

**Published:** 2025-09-13

**Authors:** Muhammad Aleem Abbas, Ga-Yeong Lee, Syed Al Jawad Sayem, Seung-Jin Lee, Seung-Chun Park

**Affiliations:** 1 Laboratory of Veterinary Pharmacokinetics, Institute for Veterinary Biomedical Science, College of Veterinary Medicine, Kyungpook National University, Daegu 41566, Republic of Korea; syedaleemabbas77@gmail.com (M.A.A.); yeong1129@knu.ac.kr (G.-Y.L.); aljawadsayem@knu.ac.kr (S.A.J.S.); 2 Developmental and Reproductive Toxicology Research Group, Korea Institute of Toxicology, Daejeon 34114, Republic of Korea; 3 Cardiovascular Research Institute, Kyungpook National University, Daegu 41566, Republic of Korea

**Keywords:** *Morus alba*, *Pinus densiflora*, pine, methyl-gallate, phytochemicals, in silico, phytotherapeutics

## Abstract

The growing challenge of antibiotic resistance and inflammation-related disorders calls for safe, multi-target therapeutic strategies. *Morus alba* (MOAL) and *Pinus densiflora* (PIDE) are known for their medicinal properties, yet their combined potential with methyl gallate (MG) has not been fully explored. In this study, the phytochemical composition of MOAL and PIDE was characterized using GC–MS, and their combined antimicrobial, antioxidant, and anti-inflammatory activities were evaluated. Hydroethanolic extracts were prepared and assessed for antioxidant activity (DPPH assay), antibacterial activity (disk diffusion, MIC, time kill), and nitric oxide (NO) suppression in Lipopolysaccharide (LPS)-stimulated macrophages, alongside MTT cytotoxicity screening. MOAL exhibited a higher extraction efficiency, reaching 500 mg/mL at 4 h, whereas *Pinus* achieved 450 mg/mL at the same time point. Both exhibited a diverse and abundant phytochemical profile. The optimized blend (MOAL:PIDE:MG, 1:1:0.1) demonstrated significantly enhanced bioactivity, with over 90% DPPH scavenging with the low IC_50_ value (66.62 mg/mL), potent inhibition of both Gram-positive and Gram-negative bacteria, and the strongest effect against *Staphylococcus aureus* (264 μg/mL). Time-kill assays confirmed rapid bactericidal action, and NO production was reduced by approximately 75% without cytotoxicity. Molecular docking identified a lead multi-target compound exhibiting strong binding affinities to COX-2, TNF-α, and Keap1, supporting its observed anti-inflammatory and antioxidant potential. These findings highlight the promise of synergistic phytochemical formulations as broad-spectrum, multifunctional therapeutic candidates, supporting further in vivo and clinical validation.

## 1. Introduction

The global rise in antimicrobial resistance (AMR), together with increasing cases of chronic inflammation and oxidative stress, underscores the urgent need for novel therapeutic agents with improved bio-efficacy and safety profiles. Conventional treatments are often limited in effectiveness, highlighting the importance of alternative approaches. Medicinal plant-derived phytochemicals, owing to their pharmacodynamic diversity and structural complexity, offer a promising strategy to combat these multifactorial health challenges. Plants naturally produce diverse bioactive compounds, including terpenoids, polyphenols, and flavonoids, which contribute to antimicrobial, antioxidant, and anti-inflammatory defense mechanisms. Ethnopharmacological evidence further supports their therapeutic potential in managing AMR and related disorders [1,2,3].

Among the promising candidates, white mulberry, or MOAL, and Korean pine, or PIDE, have been independently validated for their antibacterial, antioxidant, and anti-inflammatory properties. The root bark of MOAL (known as Sangbaekpi in traditional Korean medicine) has been used for centuries to alleviate respiratory infections, metabolic disorders, and inflammatory conditions [4]. Phytochemical analyses reveal that MOAL extracts contain prenylated flavonoids, alkaloids (e.g., moracins), and stilbenes, compounds that exhibit substantial antibacterial activity against pathogens such as *S. aureus* and *Pseudomonas aeruginosa*, including drug-resistant strains [5]. These compounds also suppress lipid peroxidation and reduce pro-inflammatory cytokine production by inhibiting NF-κB and MAPK signaling pathways [6,7]. Similarly, PIDE extracts are widely recognized in both Korean and global herbal medicine for their antibacterial, antioxidant, and anti-inflammatory properties [8,9]. Resinous compounds in PIDE, such as the terpene α-pinene, interfere with catechins and procyanidins, exerting bacteriostatic effects by disrupting bacterial membrane integrity and inhibiting biofilm formation [9,10]. PIDE extracts are potent ROS scavengers and anti-inflammatory agents, with studies having shown that an ethanol needle extract dose-dependently lowered intracellular ROS, malondialdehyde, NO, TNF-α, IL-1β, and PGE_2_ in LPS-activated macrophages, implying downregulation of iNOS/COX-2. The previous literature reports show that PIDE needle extract suppressed iNOS, IL-6, and IL-1β (via STAT1/3) in LPS-challenged macrophages. Thus, PIDE compounds act as antioxidants and modulate inflammatory mediators [11,12]. MG is a low-molecular-weight phenolic compound widely distributed in various medicinal plants. MG has garnered significant attention for its potent bactericidal properties, driven by its ability to interact with bacterial membrane proteins, disrupt cell wall permeability, and inhibit DNA gyrase and replication enzymes [13]. Moreover, MG exhibits strong radical-scavenging capacity—neutralizing DPPH, ABTS, and hydroxyl radicals—and mitigates inflammation by inhibiting STAT3 phosphorylation, which downregulates pro-inflammatory cytokines such as IL-6, TNF-α, and IL-1β [14].

The pharmacological potential of MOAL, PIDE, and MG underscores the importance of exploring these natural compounds as vital therapeutic agents against antimicrobial resistance, oxidative stress, and chronic inflammatory diseases. Despite the well-documented therapeutic potential of MOAL, PIDE, and MG, a significant gap remains in research on their combined pharmacological effects. The strategic use of synergistic phytocompounds offers a promising approach to enhancing treatment outcomes while minimizing side effects. It is reported that combining phytochemicals enhances efficacy against resistant pathogens, oxidative damage, and chronic inflammation. A study demonstrated that MOAL prenylflavonoids act synergistically with multiple drugs to reverse resistance [15]. More generally, multiple combinations can synergistically attenuate chronic inflammation by targeting multiple pathways (increasing bioavailability, antioxidant capacity, complementary signaling) [16,17]. These findings collectively highlight the need for an investigation into the combined pharmacological actions of MOAL, PIDE, and MG, particularly through chemical profiling and functional assays to elucidate their synergistic therapeutic potential.

Therefore, this study aims to comprehensively chemically profile the extracts by employing GC–MS analysis to profile the bioactive compounds in MOAL and PIDE extracts and evaluate the antibacterial, antioxidant, and anti-inflammatory properties of MOAL and PIDE extracts, both individually and in combination with MG. Furthermore, the synergistic effects of these extracts combined with MG on antimicrobial, antioxidant, and anti-inflammatory activities are evaluated in this study. We hypothesized that (1) MOAL and PIDE extracts could exhibit significant individual bioactivities against microbial growth, oxidative stress, and inflammatory responses; (2) MG could enhance these effects when combined with either extract, producing a synergistic effect greater than the sum of their actions; (3) the constituents identified via GC–MS analysis will correlate with the observed biological activities, thereby elucidating the underlying mechanisms of synergy. The findings of this study could validate the complementary interactions among these natural compounds that can contribute to the development of multi-targeted therapeutics derived from plant sources.

## 2. Materials and Methods

### 2.1. Chemicals, Media, and Reagents

All chemicals and reagents used in this study were of analytical or cell culture grade. Ethanol was obtained from Duksan (Ansan, Republic of Korea). Bacterial culture media were sourced from BD Bacto™ (Becton, Dickinson and Company, Franklin Lakes, NJ, USA). 2,2-Diphenyl-1-picrylhydrazyl (DPPH) was purchased from Sigma-Aldrich (St. Louis, MO, USA). Dulbecco’s Modified Eagle Medium (DMEM), used for cell culture experiments, was freshly procured from Welgene Inc. (Gyeongsan, Republic of Korea). The bioactive compound, MG, was sourced from Tokyo Chemical Industry Co., Ltd. (TCI, Tokyo, Japan).

### 2.2. Extraction of Bioactive Compounds from Morus alba and Pinus densiflora

Dried MOAL wood and PIDE needles were obtained from a traditional herbal market in the Republic of Korea and processed following the previously described protocol [18]. MOAL wood and PIDE needles were ground into powder and extracted using 50% ethanol (*v*/*v*) at a plant-to-solvent ratio of 1:10 (*w*/*v*) and heated using a heating mantle at 80 °C. Aliquots were collected at different time intervals (0, 1, 2, 3, and 4 h) for Brix percentage and yield analysis. The filtered extract was concentrated using a rotary vacuum evaporator (Buchi R-114, BÜCHI Labortechnik AG, Flawil, Switzerland) at a temperature of 90◦C. The concentrated extract was freeze-dried using a vacuum freeze-dryer (BioTron, Gangneung, Republic of Korea) at −55 °C and 0.04 mbar for 72 h. The ATAGO PAL-1 refractometer (Tokyo, Japan) was used to measure Brix values, according to the directions of the manufacturer, to assess the sugar content of MOAL and PIDE.

### 2.3. Gas Chromatography–Mass Spectrometry Analysis of Morus alba and Pinus densiflora Extracts

GC/MS analysis of the MOAL and PIDE extracts was conducted at the Center for Scientific Instruments, Kyungpook National University, Republic of Korea. The analysis was conducted using an HP 6890 Plus gas chromatograph and an HP 5973 mass selective detector (Hewlett-Packard, Palo Alto, CA, USA). The sample was diluted at a 1:1000 (*v*/*v*) ratio using HPLC-grade dichloromethane to ensure optimal injection concentration and chromatographic performance. A 1 µL aliquot of the diluted sample was injected into an HP-5 capillary column in splitless mode to maximize compound detection. The GC oven temperature was initially held at 50 °C for 4 min, then increased at 4 °C/min to a final temperature of 280 °C, where it was maintained for an additional 2 min. High-purity helium (99.99%) was used as the carrier gas at a constant flow rate of 0.7 mL/min to ensure efficient separation and consistent peak resolution. The mass scan range was set to *m*/*z* 40–600, and compound identification was performed using the NIST 2017 Mass Spectral Library. Quantification of individual constituents was performed using the area normalization method, which calculates the relative percentage of each compound based on its peak area in the total ion chromatogram, allowing semi-quantitative profiling when authentic standards are unavailable.

### 2.4. 2,2-Diphenyl-1-picrylhydrazyl Radical Scavenging Activity of Morus alba, Pinus densiflora, and Methyl Gallate Combinations

The antioxidant activity of MOAL, PIDE, and MG extracts, both individually and in combination (Table 1), was evaluated using the 2,2-diphenyl-1-picrylhydrazyl (DPPH) free radical scavenging assay, as previously described [19], with slight modifications. Ascorbic acid was employed as a standard for the assay. Different concentrations of MOAL, PIDE, and MG, both individually and in combination, were added to the 0.1 mmol/L DPPH solution. Absorbance was measured at 517 nm using a spectrophotometer called EPOCH™-2 (BioTek Instruments, Seoul, Republic of Korea). Radical scavenging activity was expressed as a percentage, and IC50 values were calculated from the graph using triplicate measurements.

### 2.5. Bacterial Strains and Culture Medium

Clinically relevant bacterial strains were selected for this study, including Gram-positive *S. aureus* (ATCC 29213), Gram-negative *E. coli* (ATCC 35218), and *S. typhi* (KTCC 2515). All strains were preserved and revived following ATCC and KTCC guidelines. Upon activation, each organism was streaked onto selective agar media—Mueller–Hinton Agar (MHA) for pathogen growth. Colonies were examined for morphological uniformity to ensure purity and identity before use in antimicrobial assays.

### 2.6. Bacterial Growth Curve Assay

The growth kinetics of *S. aureus*, *E. coli*, and *S. typhi* were evaluated using a growth curve assay based on viable cell counts. Purified strains were inoculated into MH Broth at two concentrations (10^5^ and 10^9^ CFU/mL) and incubated aerobically at 37 °C with agitation. Samples were aseptically collected at 0, 1, 2, 4, 8, 12, and 24 h, serially diluted in sterile saline, and plated in duplicate on MHA. After 18–24 h of incubation, colonies were counted to determine CFU/mL, and growth curves were plotted to illustrate bacterial growth phases.

### 2.7. Disk Agar Diffusion Assay

The antibacterial activity of MOAL, PIED extracts, and MG against *S. aureus*, *E. coli*, and *S. typhi* was evaluated using the disk diffusion method [20]. MH Agar plates were seeded with standardized bacterial suspensions (10^6^ CFU/mL), and sterile 6 mm disks impregnated with each test compound were placed on the surface. Following 18–24 h of incubation at 37 °C, inhibition zones were measured in millimeters. Subsequently, combinations of each extract with MG were tested on the same plates by placing disks at distances corresponding to their individual inhibition radii. Interactions were classified based on changes in inhibition zones: synergism (enhanced or merged zones), indifference (no change), or antagonism (reduced or interrupted zones) compared to those of individual treatments. The gentamicin, ciprofloxacin, and chloramphenicol were used as positive control antibiotics for *E. coli*, *S. aureus*, and *S. typhi*, respectively.

### 2.8. Antibacterial Activity of Morus alba, Pinus densiflora, and Methyl Gallate: Individual and Combinations

The minimum inhibitory concentration (MIC) and minimum bactericidal concentration (MBC) of MOAL, PIDE, and MG, both individually and in combination, were determined using the broth microdilution method [21]. Serial two-fold dilutions were prepared in MHB in 96-well plates, followed by inoculation with 10^5^ CFU/mL of bacterial suspension. After 18 h of incubation at 37 °C, bacterial growth was assessed through measuring optical density at 600 nm. The MIC was defined as the lowest concentration with showed no visible growth. For the MBC, aliquots from MIC-negative wells were plated on MHA and incubated overnight. The absence of colony formation was recorded as the MBC.

### 2.9. Time-Kill Activity of Morus alba, Pinus densiflora, and Methyl Gallate: Individual and Combinations

The time-kill assay was performed with minor modifications to established protocols [21]. *S. aureus*, *E. coli*, and *S. typhi* were grown in Mueller-Hinton Broth (MHB) at 37 °C to reach the logarithmic phase, then adjusted to a final concentration of 10^5^ CFU/mL. Each strain was treated with MOAL and PIDE extract, and MG at 1/2×, 1×, 2×, and 4× MIC. Cultures were incubated at 37 °C, and samples were collected at 0, 1, 2, 4, 6, 12, and 24 h. The samples were serially diluted in sterile saline and plated on MHA. After 48 h of incubation, the CFU/mL was determined. Time-kill curves were generated to assess the bactericidal or bacteriostatic effects of each treatment over time. Bactericidal activity was defined as a ≥3 log_10_ CFU/mL reduction relative to the initial inoculum within 24 h, following CLSI guidelines.

### 2.10. Cell Culture

RAW 264.7 murine macrophage obtained from the Korean Cell Line Bank (KCLB), Seoul, Republic of Korea, were cultured in DMEM supplemented with 10% fetal bovine serum and 1% penicillin–streptomycin. Cells were incubated overnight at 37 °C in a humidified atmosphere containing 5% CO_2_. Confluency and cell morphology were assessed under an inverted microscope. Cell density was determined using a hemocytometer, with 10 μL of cell suspension loaded and counted according to standard protocols. In total, 1 × 10^5^ cells/well were seeded into 24-well plates and allowed to adhere for 24 h under standard culture conditions.

### 2.11. MTT Assay for Cytotoxicity

Cell viability in response to individual extracts and their combinations was assessed via the MTT assay, as previously described [21]. In brief, RAW 264.7 cells were treated with test compounds for 24 h, after which 20 μL of MTT solution (5 mg/mL in PBS) was added to each well. Plates were incubated at 37 °C for 4 h in the dark. Following incubation, 200 μL of DMSO was added to dissolve the formazan crystals, and the plates were gently shaken for 10 min. Absorbance was measured at 570 nm using a microplate reader. Viability was calculated relative to untreated control cells.

### 2.12. Nitric Oxide Production Assay

The anti-inflammatory potential of the samples was evaluated by measuring nitric oxide (NO) production in lipopolysaccharide (LPS)-stimulated RAW 264.7 cells, as previously described [22]. Cells (1 × 10^5^/well) were seeded in 24-well plates and cultured for 24 h until reaching 80% confluence. After a 30 min pre-treatment with test extracts and combinations, LPS was added to induce inflammation, and cells were incubated for an additional 18 h. Supernatants were collected, and NO levels were quantified using the Griess reagent (1:1 mixture of Solutions A and B). After 10 min of incubation at room temperature, absorbance was measured at 540 nm. Nitrite concentrations were calculated using a standard curve generated from sodium nitrite.

### 2.13. In Silico Pharmacokinetic and Toxicity Analysis

All phytocompounds identified through GC–MS were retrieved from the PubChem database (https://pubchem.ncbi.nlm.nih.gov/, accessed on 16 May 2025) in SMILES format and evaluated using SwissADME (http://www.swissadme.ch/, accessed on 16 May 2025) and pkCSM (https://biosig.lab.uq.edu.au/pkcsm/, accessed on 16 May 2025) web servers. SwissADME predicted physicochemical properties, gastrointestinal (GI) absorption, bioavailability scores, BOILED-Egg BBB permeability, PAINS alerts, and drug-likeness based on Lipinski’s Rule of Five. pkCSM was used to model pharmacokinetic behavior and assess toxicity endpoints, including Ames mutagenicity, hERG I/II inhibition, acute oral toxicity (rat LD_50_), and hepatotoxicity. Compounds breaching multiple Lipinski criteria or showing high predicted toxicity were excluded from further consideration. This integrated computational approach provided a rapid, reliable screen for bioactive candidates with favorable PK profiles and acceptable safety margins.

### 2.14. Molecular Docking

#### 2.14.1. Preparation of Proteins

The three-dimensional (3D) structures of four target proteins, Cyclooxygenase-2 (COX-2, PDB ID: 5F19) [23], Tumor Necrosis Factor-alpha (TNF-α, PDB ID: 2AZ5) [24], UDP-*N*-acetylglucosamine enolpyruvyl transferase (MurA, PDB ID: 1UAE) [25], and DNA Gyrase subunit B (PDB ID: 5MMN) [26] were obtained from the Protein Data Bank (PDB) (https://www.rcsb.org/; accessed on 1 November 2020). These proteins were selected based on their critical roles in various biological processes: COX-2 in inflammation, TNF-α in cytokine signaling, MurA in bacterial cell wall synthesis, and DNA gyrase in DNA replication. Protein preprocessing and optimization were performed using the Protein Preparation Wizard in Schrödinger Maestro software version 11.1 [27]. The structures were refined at a physiological pH of 7.0, with energy minimization executed using the OPLS3 force field. The most probable ligand-binding pockets were identified using the PockDrug server, an online tool designed to predict druggable binding sites, and the receptor grid was generated accordingly for each protein.

#### 2.14.2. Ligand Preparation

The chemical structures of six selected ligands, Scopoletin, Guaiacol, Resorcinol, Furaneol, Furfural, and Benzyl alcohol, were retrieved from the PubChem compound database (https://pubchem.ncbi.nlm.nih.gov/, accessed on 23 May 2025). Each ligand was downloaded in SDF format for further processing [27]. Ligand preparation was carried out using the LigPrep module of the Schrödinger Maestro suite (version 11.1). The preparation protocol involved the generation of all possible ionization and tautomeric states at a target pH of 7.0 ± 2.0, ensuring proper protonation for physiological conditions. The OPLS3 force field was applied to optimize the molecular geometries and minimize their energy states, producing low-energy, 3D-optimized ligand conformations suitable for docking studies [28].

#### 2.14.3. Glide Standard Precision Docking

Molecular docking studies were performed to predict the potential binding modes and interaction mechanisms of the selected ligands with their respective protein targets, and to compare their affinities with those of reference compounds. The docking process was conducted using the Ligand Docking tool in the Schrödinger Maestro suite (version 11.1) [27], employing the Glide Standard Precision (SP) algorithm. Each prepared ligand was docked into the receptor grid generated for the respective proteins. The docking protocol considered flexibility in the ligand while keeping the receptor rigid. The resulting docking scores and interaction data were compiled in Maestro-generated spreadsheets for post-docking analysis. For detailed visualization and interpretation of molecular interactions, including hydrogen bonds, hydrophobic contacts, and π–π stacking, BIOVIA Discovery Studio Visualizer was used to generate high-resolution 2D and 3D interaction diagrams.

## 3. Results

### 3.1. Extraction Kinetics of Yield and Brix%

**Figure 1:**
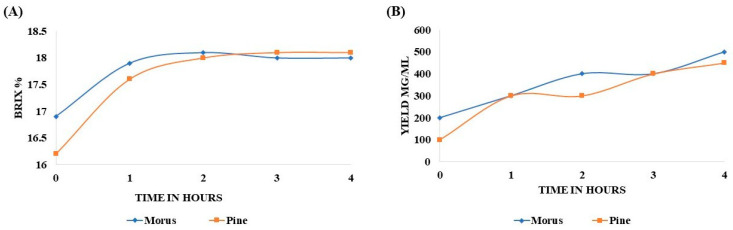
Variation in Brix percentage of MOAL and PIED extracts over a 4 h extraction period using 50% ethanol (*v*/*v*) at a 1:10 solid-to-solvent ratio (**A**). Extract yields (mg/mL) of *Morus* and PIED as a function of extraction time under identical conditions. Data represent mean values from replicate extractions (**B**). MOAL, *Morus alba*; *PIED*, *Pinus densiflora*.

The Brix percentage of MOAL extract increased from 16.9% to 17.9% after 1 h, peaked at 18.2% after 2 h, and remained stable thereafter. In contrast, the PIDE extract steadily increased from 16.1% to 17.6% in the first hour, attaining 18.1% by the second hour. No significant changes were observed between the second and fourth hour (Figure 1A). The extractability of MOAL consistently exceeded that of PIED throughout the 4 h extraction (Figure 1B and Table 2). Initially, MOAL yielded 200 mg/mL, twice the yield of PIDE (100 mg/mL). Both extracts reached 300 mg/mL by the end of the first hour, indicating the rapid solubilization of readily extractable constituents. However, after 2 h, the MOAL yield continued to rise, reaching 500 mg/mL at the fourth hour, while PIDE plateaued at 300 mg/mL before gradually increasing to 450 mg/mL by the end of the process.

### 3.2. Gas Chromatography–Mass Spectroscopy Profiling of Morus alba Extract

**Figure 2:**
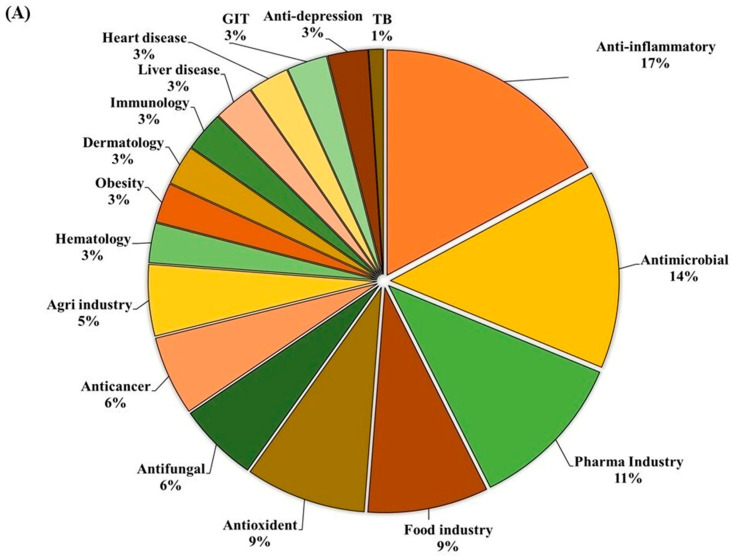

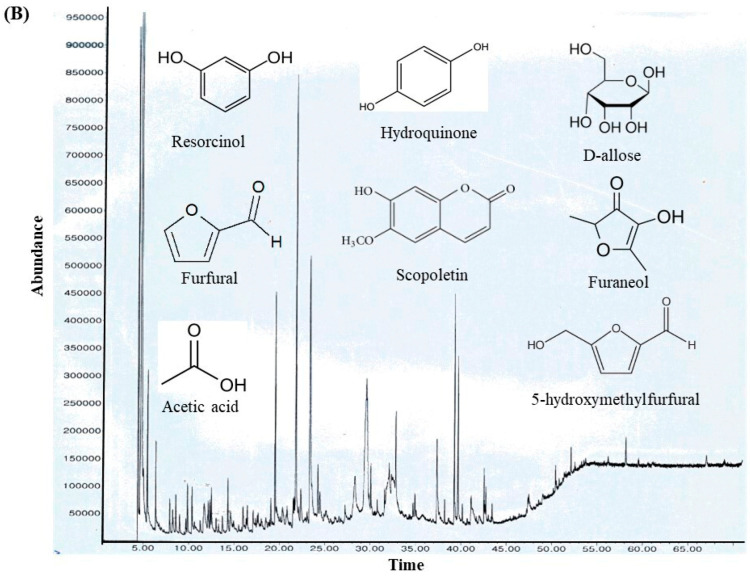
(**A**) GC–MS analysis of MOAL chemicals, categorizing the detected bioactive compounds by their reported biological activities. The pie chart illustrates the relative abundances of compounds with activities such as antibacterial, antioxidant, and anti-inflammatory effects, among others. Each colored segment represents a specific biological function, with the percentage indicating its proportional contribution to the extract. This comprehensive profile highlights the multifunctional therapeutic potential of MOAL phytochemicals and provides a basis for understanding their synergistic roles in both traditional and modern medicinal applications. (**B**) The chromatograms generated from the GC/MS analysis of MOAL show peaks corresponding to specific compounds obtained at a specific RT. The chemical structures of the bioactive compounds, along with their RT, are also provided. GC–MS, Gas chromatography–mass spectrometry; MOAL, *Morus alba;* RT, retention time.

GC-MS analysis of the MOAL extract revealed a variety of bioactive compounds, including phenolics, furans, fatty acids, rare sugars, and heterocycles (Table 3; Figure 2). Among the phenolics, resorcinol (10.47%) was the dominant compound, known for its antimicrobial and antiseptic properties [29,30]. Hydroquinone (0.38%) was also identified, known for its antioxidant activity. The extract was rich in furan derivatives, including 5-hydroxymethylfurfural (10.27%), furfural (1.06%), and 4H-pyran-4-one (4.34%), all of which are known for their antioxidant and anti-inflammatory effects [31,32]. A high concentration of D-allose (13.54%) was detected, a rare sugar known for its antiproliferative and cryoprotective properties. Palmitic acid (3.45%) showed anti-inflammatory and immunomodulatory potential, while scopoletin (3.23%) exhibited antibacterial and anti-inflammatory activity [33,34]. Minor components, such as furaneol (0.58%) and 4-methoxy-5H-furan-2-one, exhibited anti-biofilm and antioxidant effects, respectively [35,36]. These findings highlight the multifunctional therapeutic potential of MOAL attributed to synergistic phytochemical interactions.

### 3.3. Gas Chromatography–Mass Spectroscopy Profiling of Pinus densiflora Extract

**Figure 3:**
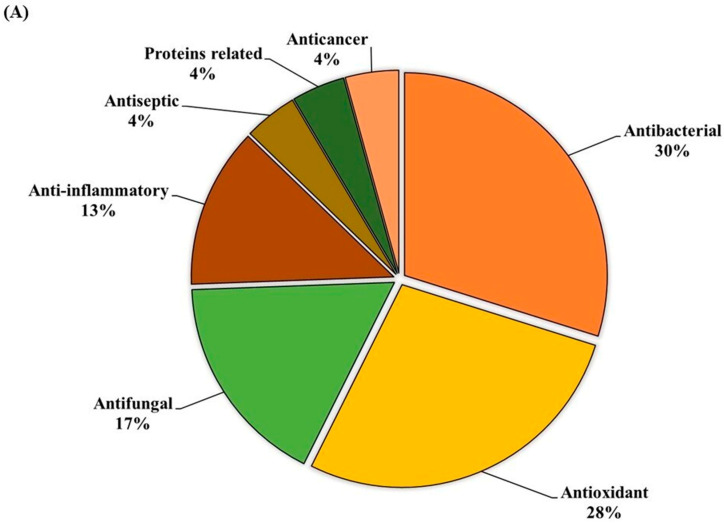

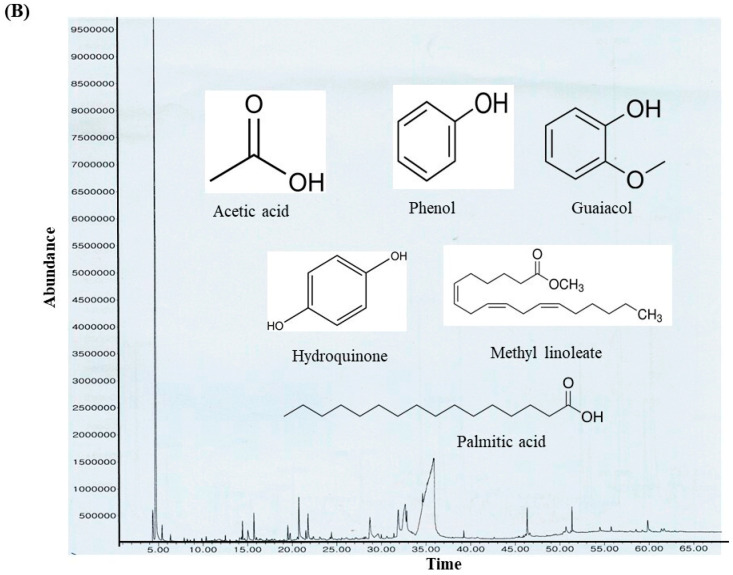
(**A**) GC–MS analysis of the PIED extract revealed the distribution of bioactive compounds based on their predicted biological activities. The pie chart segments represent the relative abundances of compounds associated with antibacterial, antioxidant, antifungal, anti-inflammatory, anticancer, antiseptic, and protein-related functionalities, highlighting the multifunctional potential of PIED-derived chemicals. (**B**) The chromatograms generated from the GC/MS analysis of PIED, each peak represents a specific compound obtained at a specific RT. The chemical structures of the bioactive compounds, along with their RT, are also provided. Gas chromatography–mass spectrometry; *Morus alba*; RT, retention time.

The ethanolic-aqueous extract of PIED revealed a diverse phytochemical profile (Table 4) with bioactivities (Figure 3). Preliminary screening identified phenolics, flavonoids, tannins, alkaloids, and terpenoids. GC–MS analysis revealed several volatile and semi-volatile compounds grouped into phenolics, acids, esters, alcohols, ketones, and hydrocarbons. Guaiacol (12%) was the most abundant phenolic, followed by phenol (9%) and hydroquinone (7%). Acetic acid (10%), palmitic acid (5%), and methyl linoleate (2%) dominated the acid and ester groups. Phytol was the main diterpenoid alcohol, while benzyl alcohol (1%), along with trace aldehydes and ketones, indicated lignin breakdown. Long-chain alkanes, such as n-hexadecane (3–4%) and trace siloxanes, were also detected. The phytochemical complexity of PIED, rich in phenolics and oxygenated volatiles, supports its therapeutic potential and aligns with the known medicinal profiles of PIED-derived extracts.

### 3.4. 2,2-Diphenyl-1-picrylhydrazyl Radical Scavenging Activity of Morus alba, Pinus densiflora, and Methyl Gallate

**Figure 4:**
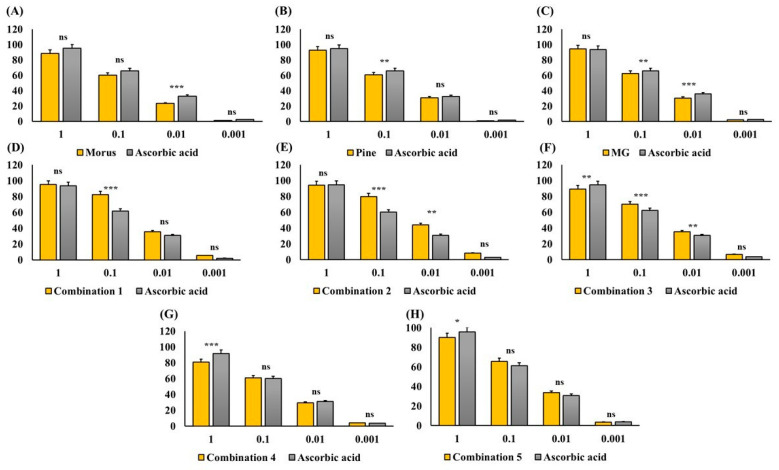
DPPH Radical Scavenging Activity of MOAL, PIED, MG, and their combinations. Each bar chart (**A**–**H**) illustrates the percentage of DPPH radical inhibition at various concentrations (1, 0.1, 0.01, and 0.001 mg/mL) for the individual extracts (*Morus*, *Pinus*, and MG) and their fixed-ratio combinations (Combinations 1–5). Ascorbic acid served as a standard reference compound (gray bars) to compare antioxidant activity. Higher percentages indicate stronger free radical scavenging capacities. Data are expressed as mean ± SD. Statistical significance compared with ascorbic acid: *p* < 0.05 (*), *p* < 0.01 (**), *p* < 0.001 (***), ns = not significant. MG, Methyl Gallate; DPPH, 2,2-diphenyl-1-picrylhydrazyl; MOAL, *Morus alba*; PIED, *Pinus densiflora*.

**Figure 5:**
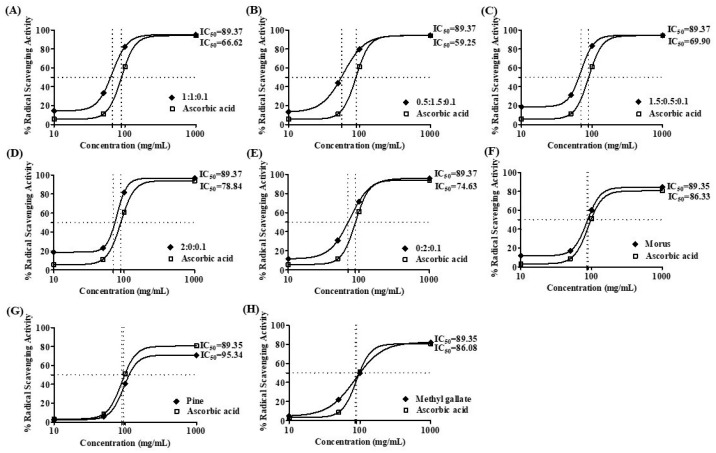
DPPH radical scavenging activity IC 50 value of MOAL, PIED, MG, and their combinations. Subfigures (**A**–**E**) represent different fixed-ratio combinations of *Morus alba*, *Pinus densiflora*, and methyl gallate, while (**F**–**H**) show the individual extracts (MOAL, PIED, and MG). The *x*-axis shows extract concentrations (mg/mL), and the *y*-axis indicates percentage DPPH radical scavenging activity. The dashed vertical and horizontal lines denote the IC_50_ values (the concentration required to achieve 50% inhibition) for each sample compared with the standard reference (ascorbic acid). IC_50_ values are presented adjacent to each curve. MG, Methyl Gallate; DPPH, 2,2-diphenyl-1-picrylhydrazyl; MOAL, *Morus alba*; PIED, *Pinus densiflora*.

The antioxidant capacity of MOAL, PIED, and MG, both individually and in five fixed-ratio mixtures, was evaluated using the DPPH radical scavenging assay (Figure 4 and Figure 5). Among the individual agents, MG exhibited the highest activity (94.27% at 1 mg/mL), closely followed by MOAL (88.61%), with both showing activities similar to ascorbic acid (95.05%). All combinations outperformed the individual components. Combination 1 (MOAL: PIED: MG = 1:1:0.1) demonstrated the highest activity (95.16%), exceeding ascorbic acid. Combination 2 (0.5:1.5:0.1) and Combination 5 (0:2:0.1) showed strong activity at 94.39% and 90.08%, respectively, highlighting the synergistic role of PIED in enhancing antioxidant effects. In contrast, Combinations 3 and 4, which lacked PIED, showed reduced activity (e.g., 80.75%), confirming that both MOAL and PIED are essential for achieving maximal antioxidant synergy. These findings highlight the enhanced efficacy of MOAL–PIED–MG combinations, particularly at a 1:1:0.1 ratio, which surpassed the standard antioxidant benchmark.

### 3.5. Growth Curve Analysis of S. aureus, S. typhi, and E. coli Under Varying Initial Inoculum Densities

**Figure 6:**
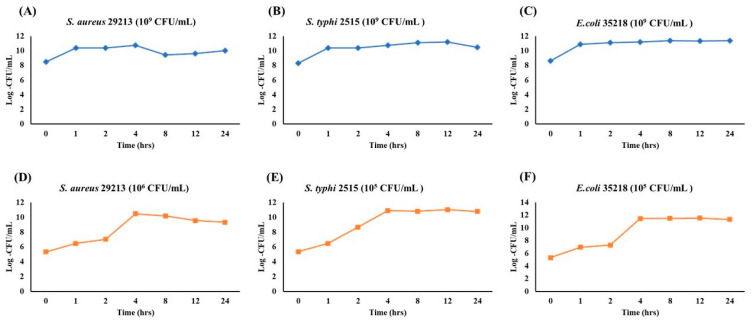
Growth Kinetics of *S. aureus*, *S. typhi*, and *E. coli* at high (10^9^ CFU/mL) and low (10^5^ CFU/mL) inoculum densities over 24 h. Each plot depicts the bacterial population (Log CFU/mL) monitored at 0, 1, 2, 4, 8, 12, and 24 h. Panels (**A**–**C**) represent the high-inoculum condition (10^9^ CFU/mL) for *S. aureus* (**A**), *S. typhi* (**B**), and *E. coli* (**C**), whereas panels (**D**–**F**) illustrate growth starting at a lower inoculum density (10^5^ CFU/mL).

To evaluate growth dynamics and optimize sampling points for future assays, the growth of *S. aureus*, *S. typhi*, and *E. coli* was monitored over 24 h at two initial inoculum levels (10^9^ and 10^5^–10^6^ CFU/mL). At a high inoculum density (10^9^ CFU/mL), all strains showed minimal growth and quickly reached saturation (Figure 6A–C). *S. aureus* (Figure 6A) entered the log phase within 1 h before plateauing. *S. typhi* (Figure 6B) rapidly reached log ~11 CFU/mL, maintaining stability. *E. coli* (Figure 6C) exhibited the fastest transition, plateauing by 2 h. At lower inoculum levels (10^5^–10^6^ CFU/mL), classic growth curve phases were observed (Figure 6D–F). *S. aureus* (Figure 6D) reached peak growth at 4 h. *S. typhi* (Figure 6E) showed early exponential growth starting at 1 h and plateaued at 4 h. *E. coli* (Figure 6F) demonstrated the strongest proliferation, reaching the stationary phase by 4 h and maintaining it through 24 h. These findings indicate that initial inoculum density significantly influences bacterial growth phase dynamics. Lower inocula facilitate clear differentiation of growth phases, making them more suitable for antimicrobial or stress-response assays.

### 3.6. Antibacterial Activity Screening by Disk Diffusion Assay

**Figure 7:**
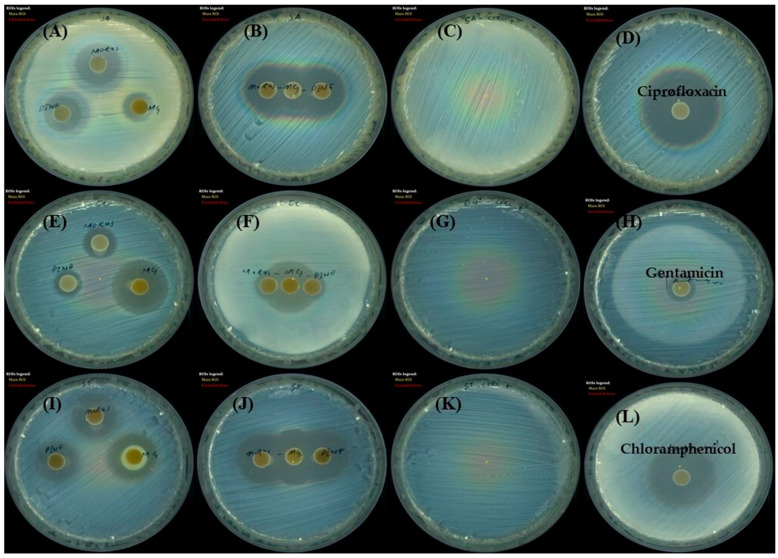
Disk diffusion assay showing the antibacterial activity of *Morus*, *Pinus*, and MG against three bacterial strains. Zones (mm) of inhibition, which indicate bacterial growth suppression, are shown for (**A**–**D**) *Staphylococcus aureus*, (**E**–**H**) *Escherichia coli*, and *Salmonella typhi*, (**I**–**L**). Each panel represents agar plates inoculated with the specified bacterium and treated disks containing either individual extracts or MG. Clear zones surrounding the disks indicate the potency of each treatment, with larger diameters corresponding to stronger antibacterial activity. MG, methyl gallate.

The antibacterial effects of MOAL, PIED, and MG were assessed using the disk diffusion method against *S. aureus*, *E. coli*, and *S. typhi* (Figure 7). MOAL exhibited the highest antibacterial activity against *S. aureus* (27.04 mm), with moderate inhibition observed against *S. typhi* (15.82 mm) and *E. coli* (12.81 mm). PIED also showed antibacterial activity against *S. aureus* (24.25 mm), with weaker inhibition observed against *S. typhi* (14.07 mm) and *E. coli* (10.56 mm). In contrast, MG exhibited the greatest antibacterial activity against *E. coli* (24.43 mm), followed by *S. typhi* (19.22 mm) and *S. aureus* (15.37 mm). Combination assays, conducted by placing MOAL, PIED, and MG disks in proximity, revealed synergistic or additive effects, particularly against *S. aureus*, where merged zones of inhibition were observed. A slight enhancement was detected against *S. typhi*, while *E. coli* showed no significant change. Overall, MOAL displayed the highest standalone efficacy against *S. aureus*, and its combination with MG enhanced activity against Gram-positive strains, indicating potential for synergistic antibacterial formulations.

### 3.7. Antibacterial Properties of Morus alba, Pinus densiflora, and Methyl Gallate Evaluation by the Dilution Method

**Figure 8:**
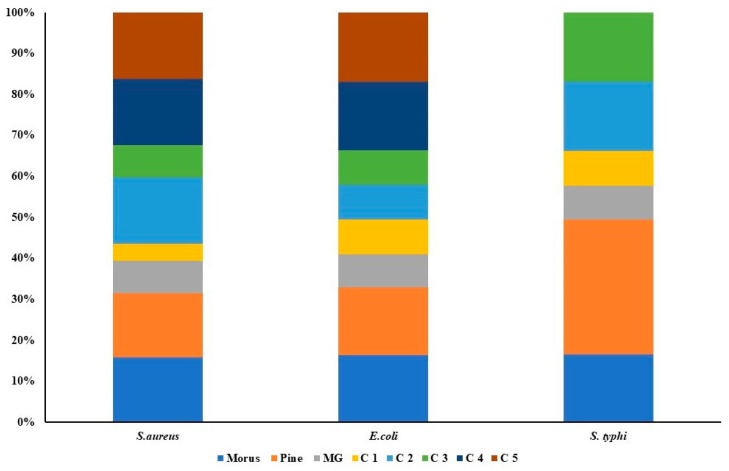
Antibacterial activity of *Morus*, *Pinus*, MG, and their five combination treatments at varying ratios. Each stacked bar represents a specific bacterial strain (*Staphylococcus aureus*, *Escherichia coli*, and *Salmonella typhi*), with colored segments indicating the relative inhibitory contributions of individual extracts (*Morus*, *Pinus*, MG) and their combination formulas (C1–C5). The overall bar height indicates the total antibacterial effect, while the size of each segment indicates the proportional effect of the agent or combination.

The antibacterial activities of MOAL, PIED, and MG were evaluated individually and in combination against *S. aureus*, *E. coli*, and *S. typhi* using MIC assays (Figure 8). MOAL and PIED individually showed moderate activity, with MICs of 1024 μg/mL against *S. aureus* and 2048 μg/mL against *E. coli*. For *S. typhi*, PIED displayed weaker efficacy, with an MIC of 4096 μg/mL. MG exhibited the strongest individual activity, particularly against *S. aureus*, with an MIC of 512 μg/mL. However, all agents required concentrations above 5 mg/mL to achieve bactericidal effects. The combination of MOAL:PIED:MG at a 1:1:0.1 ratio significantly enhanced antibacterial efficacy, reducing MIC values to 264 μg/mL for *S. aureus* and 1050 μg/mL for *E. coli* and *S. typhi*. The 1.5:0.5:0.1 ratio, with higher MOAL content, showed the most potent activity among all tested formulations. These findings indicate a synergistic interaction, particularly against *S. aureus*, positioning the MOAL–PIED–MG combination as a promising candidate for enhanced antibacterial therapy.

### 3.8. Time-Killing Activity of Morus alba, Pinus densiflora, Methyl Gallate, and Their Combinations

**Figure 9:**
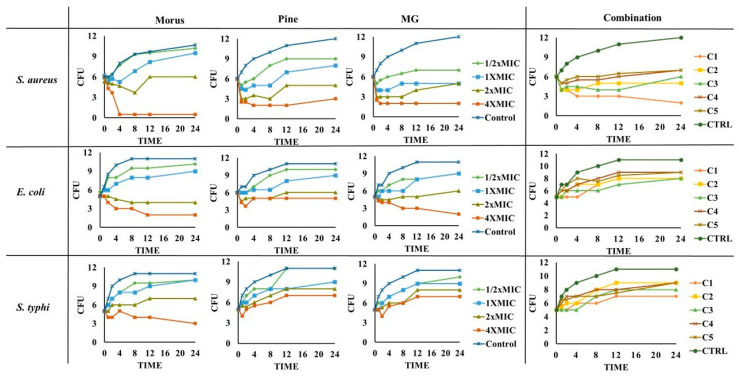
Time-kill curves for MOAL, PIED, MG, and their five combination ratios (C1–C5) against *S. aureus*, *E. coli*, and *S. typhi*. Each panel presents changes in bacterial counts (CFU/mL) over 24 h following treatment at varying concentrations (0–4 × MIC). Samples were collected at specified time intervals and plated to quantify surviving bacteria. Curves that drop to or near zero indicate effective bactericidal activity, while curves remaining above the initial inoculum level suggest reduced or no antimicrobial effect. The comparative analysis highlights potential synergistic or additive effects when phytochemicals from *Morus* and *Pinus* are combined with MG. MG, methyl gallate.

The standard time-kill assay for natural products [86] demonstrated variable antibacterial activity of MOAL, PIED, and MG against *S. aureus*, *E. coli*, and *S. typhi* (Figure 9).

MOAL exhibited the strongest bactericidal effect against *S. aureus*, achieving complete elimination at 4 × MIC within 4 h and maintaining suppression up to 24 h. Against *E. coli*, MOAL showed moderate activity, requiring higher concentrations (>2 × MIC) for significant reductions, while activity against *S. typhi* remained limited, with CFU decreases of <1 log_10_ even at 4 × MIC. PIED demonstrated dose-dependent bacteriostatic activity, particularly against *S. aureus*, but did not achieve complete bactericidal outcomes at any tested concentration. MG displayed the highest overall potency, reducing *S. aureus* and *E. coli* counts by >3 log^10^ CFU at 4 × MIC after 24 h, while showing moderate inhibition of *S. typhi*. Combination treatments (C1–C5) enhanced activity exclusively against *S. aureus*, where C1 reduced bacterial load from 6 to 2 log^10^ CFU after 24 h, meeting the accepted bactericidal threshold (≥3 log_10_ CFU reduction). For *E. coli* and *S. typhi*, all combinations demonstrated only bacteriostatic effects with CFU reductions <3 log_10_, indicating limited synergy against these Gram-negative strains.

### 3.9. Cytotoxic Effects of Morus alba, Pinus densiflora, Methyl Gallate, and Combinations of the Three

**Figure 10:**
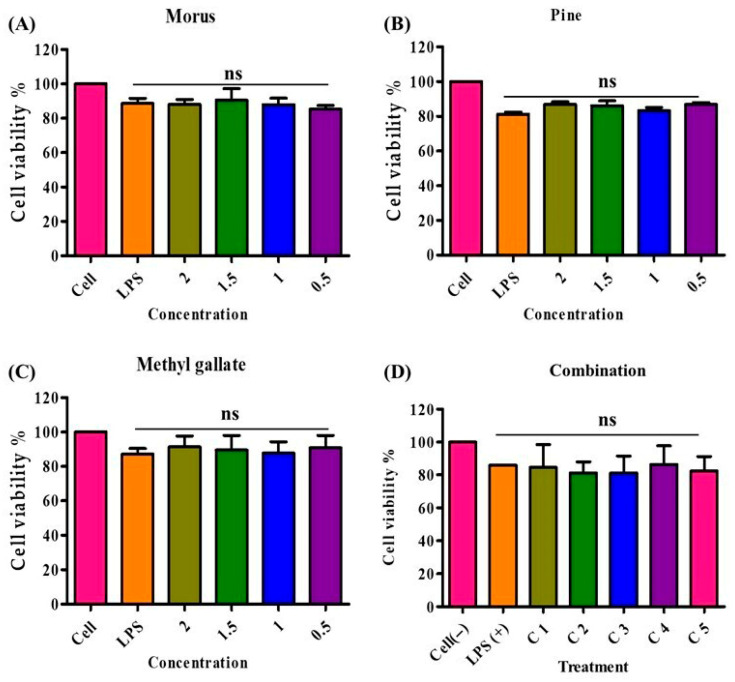
Cell viability of RAW 264.7 macrophages treated with *Morus*, PIED (*Pinus*), MG, and their various combinations. Panels (**A**–**C**) show the percentage of viable cells after 24 h of exposure to increasing concentrations (0.5–2 mg/mL) of each extract or compound, while Panel (**D**) illustrates cell viability across five different combination ratios (C1–C5). An LPS-stimulated control group (LPS+) represents the inflammatory condition, and an untreated control group (Cell) serves as the baseline. All treatments maintained cell viability at or above 80%, indicating low cytotoxicity of these natural extracts and their synergistic mixtures. PIED, *pinus densiflora*; MG, methyl gallate; LPS, lipopolysaccharide.

The cytotoxicity of MOAL, PIED, and MG was evaluated in RAW 264.7 macrophages using the MTT assay (Figure 10). Treatments were applied individually and in combination across a range of concentrations, with the highest tested dose at 2000 µg/mL. All individual treatments demonstrated high cellular compatibility, with MOAL maintaining 83–97% cell viability, PIED 81–88%, and MG 81–98%. No formulation reduced viability below 80%, even at the maximum concentration tested. All five MOAL–PIED–MG combinations similarly preserved cell viability above 80%, confirming the noncytotoxic nature of both the individual and combined treatments. These findings indicate their safety for further biological and therapeutic applications.

### 3.10. Nitric Oxide Inhibition Activity of Morus alba, Pinus densiflora, Methyl Gallate, and Their Combinations

**Figure 11:**
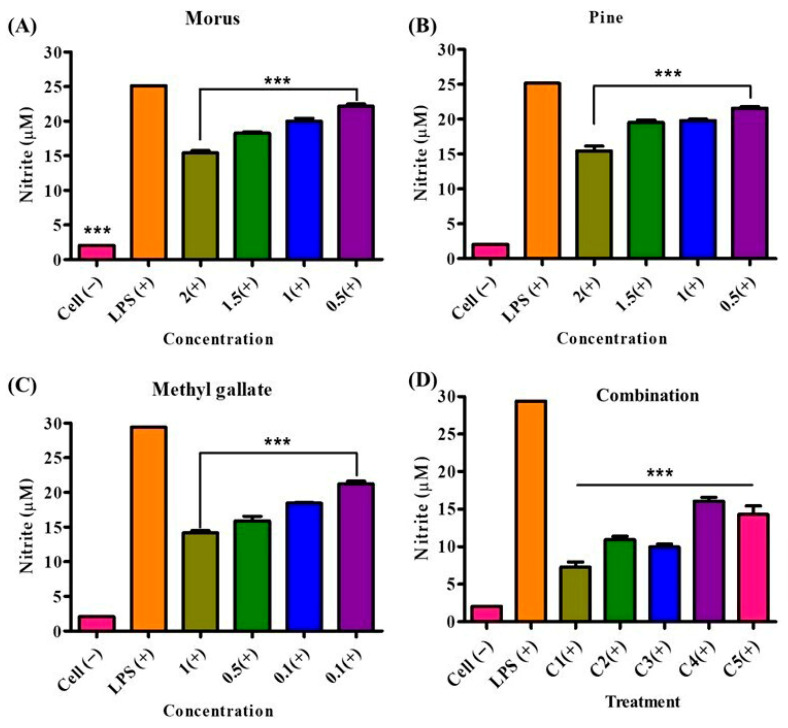
NO inhibition by *Morus* (**A**), *Pinus* (**B**), methyl gallate (**C**), and their combination (**D**) in LPS-stimulated RAW 264.7 cells. RAW 264.7 macrophages were pretreated with individual extracts or their mixtures at the indicated concentrations, followed by LPS stimulation to induce NO production. Nitrite levels (μM) in culture supernatants were quantified using the Griess assay. “Cell (−)” indicates untreated controls, while “LPS (+)” denotes LPS-only stimulation. All treatments significantly reduced nitrite accumulation compared with the LPS-only group (*** *p* < 0.001). Combination treatment elicited the strongest suppression, suggesting potential synergistic anti-inflammatory activity at higher concentrations. These findings highlight enhanced NO-suppression by combined phytochemical treatments. NO, nitric oxide; PIED, Pinus densiflora; MG, methyl gallate; LPS, lipopolysaccharide.

The inhibitory effects of MOAL, PIED, and MG on LPS-induced NO production in RAW 264.7 macrophages were evaluated individually and in combination (Figure 11). All treatments significantly reduced NO levels compared to those of the LPS-stimulated control, exhibiting concentration-dependent responses. MOAL showed concentration-dependent inhibition, achieving a maximum reduction of 37.2% at 2 mg/mL. Lower doses (1.5, 1.0, and 0.5 mg/mL) resulted in 25.6%, 22.4%, and 10.1% inhibition, respectively. PIED followed a similar trend, with peak inhibition of 39.8% at 2 mg/mL and moderate effects at lower concentrations (15.2–21.1%). MG demonstrated potent activity at lower doses, achieving 52.4% inhibition at 1 mg/mL and 25.2–33.0% inhibition at 0.1 mg/mL. Among the combinations, Combination 1 (MOAL:PIED:MG = 1:1:0.1) exhibited the strongest inhibitory effect, reducing NO production by 75.1%. Combinations 2, 3, and 5 showed consistent inhibition, ranging from 63.3% to 64.9%, while Combination 4 achieved 44.1%. These findings indicate a synergistic inhibitory effect in the combined treatments, particularly in Combination 1, which markedly outperformed the individual components.

### 3.11. In Silico Pharmacokinetic, Toxicity, and Bioavailability Profiling of *Morus alba* and Pinus densiflora Selected Phytochemicals

In silico pharmacokinetic and toxicity profiling of ten MOAL and PIED phytochemicals (Table 5) indicated broadly favorable oral drug-likeness characteristics. All compounds satisfied the principal Lipinski criteria (molecular weight < 500 g/mol, hydrogen bond acceptors < 10, hydrogen bond donors < 5, and log P < 5). Veber descriptors were also within acceptable limits (TPSA < 140 Å^2^, nRB < 10) for most compounds; however, long-chain lipids such as palmitic acid, methyl linoleate, phytol, and n-hexadecane exceeded the recommended flexibility threshold. Predicted gastrointestinal (GI) absorption was high for 19 of the 20 molecules, with human intestinal absorption (HIA) values between 86% and 100%, and bioavailability scores of either 0.55 or 0.85. D-allose was the sole exception, exhibiting low predicted GI absorption (21.51%) due to high polarity (TPSA = 110.38 Å^2^) and a high hydrogen bond donor count. No pan-assay interference structure (PAINS) alerts were detected for any compound. All molecules were predicted to be non-hepatotoxic and devoid of hERG I/II cardiotoxicity liabilities. Ames mutagenicity predictions were negative for all except furfural, which showed a positive result despite excellent permeability (HIA = 100%, bioavailability score = 0.55). Predicted acute toxicity (rat LD_50_) ranged from 1.214 to 2.429 mol/kg, with D-allose and furfural representing the lowest and highest toxicity estimates, respectively.

**Figure 12:**
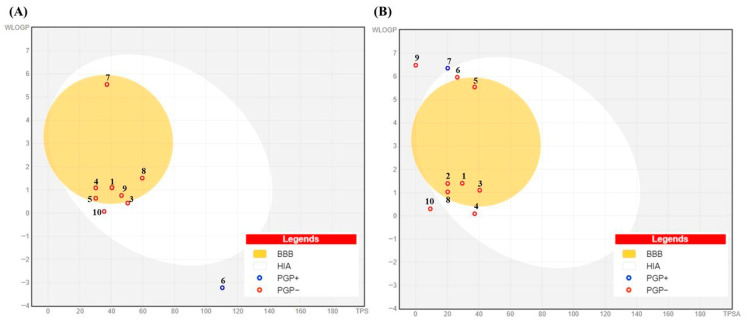
BOILED-Egg model for (**A**) *Morus alba* and (**B**) *Pinus* phytochemicals showing predicted gastrointestinal absorption (HIA, white region) and blood–brain barrier penetration (BBB, yellow region) based on WLOGP and TPSA. Red circles (PGP−) indicate compounds not predicted to be P-glycoprotein substrates; blue circles (PGP+) indicate predicted substrates. Numbers in the figure correspond to the compound numbers listed in Table 5.

BOILED-Egg analysis (Figure 12) revealed that most MOAL and PIED phytochemicals fall within the high gastrointestinal absorption (HIA, white region) or blood–brain barrier (BBB, yellow region) zones, indicating a favorable balance between polarity and lipophilicity. Several compounds also showed BBB penetration potential, while many were predicted to be non-P-glycoprotein substrates (PGP−), supporting their potential for efficient absorption and, where relevant, CNS accessibility. Scopoletin, guaiacol, and resorcinol demonstrated favorable LogP values (2.1, 1.8, and 0.9, respectively) and high GI absorption (>90%). BOILED-Egg models predict BBB penetration for scopoletin and guaiacol but not resorcinol, indicating potential CNS activity for the first two. These findings further strengthen the oral drug-likeness profile predicted by SwissADME, aligning with the high bioavailability scores and clean safety profiles described earlier.

### 3.12. Molecular Docking Study

**Figure 13:**
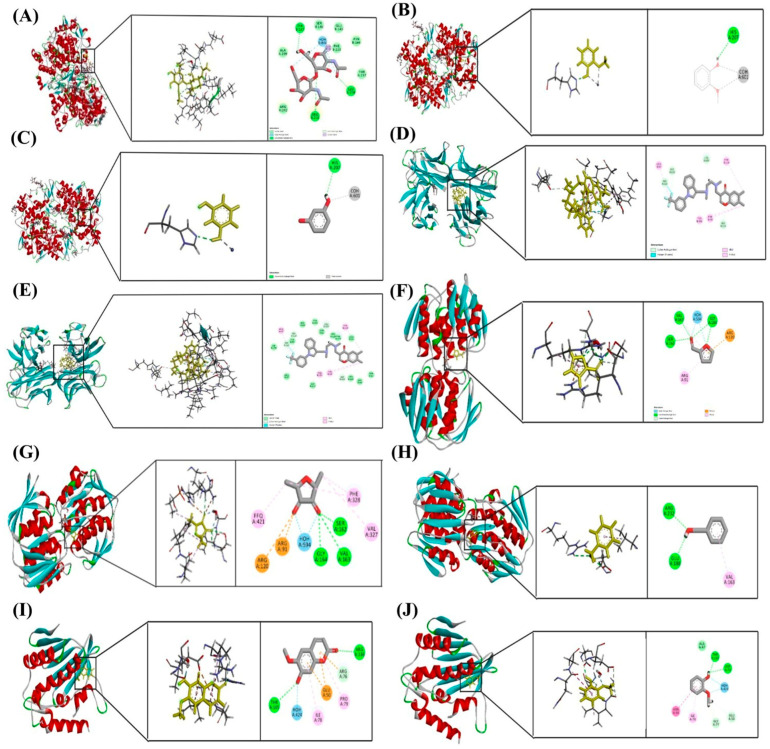
Molecular docking poses of selected phytochemicals with key protein targets. (**A**) Scopoletin bound to COX-2 (PDB ID: 5F19); (**B**) Guaiacol bound to COX-2 (5F19); (**C**) Resorcinol bound to COX-2 (5F19); (**D**) Scopoletin bound to TNF-α (2AZ5); (**E**) Furaneol bound to TNF-α (2AZ5); (**F**) Furfural bound to MurA (1UAE); (**G**) Furaneol bound to MurA (1UAE); (**H**) Benzyl alcohol bound to MurA (1UAE); (**I**) Scopoletin bound to Keap1 (5MMN); (**J**) Guaiacol bound to Keap1 (5MMN). Each docking pose highlights the orientation of the ligand within the active site and the key interactions stabilizing the ligand–protein complex.

This molecular docking study evaluated phytochemicals, Scopoletin, Guaiacol, Resorcinol, Furaneol, Furfural, and Benzyl alcohol, derived from MOAL and PIED extracts, against key protein targets involved in inflammation, immune regulation, and bacterial pathogenesis. The selected protein targets included cyclooxygenase-2 (COX-2), tumor necrosis factor-alpha (TNF-α), DNA gyrase, UDP-*N*-acetylglucosamine enolpyruvyl transferase (MurA), Kelch-like ECH-associated protein 1 (Keap1), and dihydrofolate reductase (DHFR), visualized in Figure 13.

Among all tested compounds, Scopoletin exhibited the most favorable binding profiles across multiple targets. It demonstrated strong affinities for COX-2 (−5.889 kcal/mol), TNF-α (−5.969 kcal/mol), and Keap1 (−5.321 kcal/mol), indicating a broad potential for modulating inflammatory and oxidative pathways. Detailed analysis of docking interactions revealed that Scopoletin engages in stable hydrogen bonding and π–π stacking within the active sites, closely resembling the binding modes of known inhibitors.

Resorcinol also showed promising binding with COX-2 (−5.840 kcal/mol), Table 6, supporting its role as an anti-inflammatory agent. Guaiacol, while exhibiting moderate binding to COX-2 (−5.429 kcal/mol), showed notable interaction with DNA gyrase (−5.171 kcal/mol), suggesting a mechanism of antibacterial action through the inhibition of DNA replication.

Furfural and Furaneol displayed moderate binding affinities towards MurA, with docking scores of −5.266 kcal/mol and −5.098 kcal/mol, respectively, indicating potential activity against bacterial cell wall biosynthesis. Benzyl alcohol showed the weakest binding to MurA (−4.721 kcal/mol), suggesting limited efficacy as an antimicrobial agent. Furaneol also docked with TNF-α (−4.689 kcal/mol), although with significantly lower affinity compared to Scopoletin.

Taken together, Scopoletin emerged as the most promising multi-target phytochemical among those tested, with favorable interactions across inflammation-related, oxidative stress, and bacterial targets. These results provide a theoretical basis for further pharmacological investigation.

## 4. Discussions

The investigation of MOAL, PIED, and MG revealed strong bioactivity driven by the synergistic effects of their phytochemical components. MOAL and PIED showed rapid extraction kinetics, typical for polyphenol-rich matrices, enabling subsequent assays to utilize preparations enriched with phytochemicals. Antioxidant-rich plant materials demonstrate high extraction efficiencies, consistent with findings from previous studies [87].

MOAL, specifically, contains a diverse range of bioactive molecules that show its extensive pharmacological potential in antibacterial, antioxidant, and anti-inflammatory treatments. Resorcinol and hydroquinone stand out among phenolic compounds for their continuous bactericidal effects, which result from microbial cell membrane disruption and enzyme activity inhibition [30]. The antioxidant properties of furan derivatives HMF and 4H-pyran-4-one stem from their ability to neutralize free radicals and inhibit lipid peroxidation, making them applicable in both the food and pharmaceutical sectors, according to Rosatella et al. and Čechovská et al. [31,32]. The presence of D-allose introduces new prospects for its application as an anticancer agent and immune system modulator [33]. Scopoletin, a phytoalexin found in medicinal plants, demonstrates anti-inflammatory and antimicrobial potential by downregulating NF-κB signaling, reducing pro-inflammatory cytokines such as IL-6 and TNF-α, and inhibiting microbial biofilm formation, according to studies by Gnonlonfin et al. [63]. Furthermore, PIED predominantly contains the phenolic compounds guaiacol, phenol, and hydroquinone. Research highlights that guaiacol possesses antioxidant, anti-inflammatory, and anticancer properties and was traditionally utilized as both an antiseptic and expectorant [88]. The compound serves as an antiseptic and local anesthetic and also as an expectorant in cough treatments [88]. Phenol maintains its strong bactericidal properties as one of the first antiseptics in medicine [89], while hydroquinone shows excellent antioxidant properties and effective antibacterial activity against *S. aureus* [90]. The discovery of 3-hydroxybenzoic acid introduces both anti-biofilm and anti-quorum-sensing properties to the antimicrobial extract, enhancing its effectiveness against *Acinetobacter baumannii* [91] and supporting antioxidant defense [92]. The combined presence of these phenolics provides essential support for the effectiveness of the extract in antimicrobial, antioxidant, and anti-inflammatory treatments. Acetic acid is an important volatile acid component with significant bactericidal properties, even in minimal concentrations, boosting the antiseptic properties of the extract [93]. The terpenoid phytol and fatty acid palmitic acid are crucial components with antimicrobial, anti-inflammatory, and antioxidant effects. Phytol demonstrates neuroprotective and anticancer properties, while palmitic acid strengthens cell membranes and aids metabolic processes [94]. Research supports previous findings that PIDE species and commercial extracts such as Pycnogenol exhibit antioxidant and anti-inflammatory effects in clinical studies [95]. The diverse phytochemical composition of PIDE extract provides various therapeutic benefits. The interaction between identified compounds in the PIDE extract indicates its potential as a natural treatment option for managing infections, oxidative stress, and inflammation.

All samples demonstrated concentration-dependent free radical scavenging activities, with MOAL + PIED treatment outperforming individual treatments. Among the tested mixtures, the balanced ratio in Combination 1 (1:1:0.1) produced the most effective antioxidant response, likely due to synergistic interactions between phenolic and flavonoid compounds from both plant sources and MG. The observed synergistic effect indicates that both components either regenerate each other or simultaneously target radical species. These enhanced antioxidant mechanisms are clinically significant due to the established relationship between oxidative stress and inflammation.

The antibacterial tests proved MOAL, PIED, and MG to be strong inhibitors, while combined treatments showed synergistic enhancement. The inhibition zones of the combined MOAL, PIED, and MG demonstrated a significant expansion against *S. aureus*, indicating a synergistic or additive effect. Combination C1 demonstrated a strong synergistic effect, significantly reducing the MIC against all tested bacterial strains compared to each single component. Time-kill kinetics reveal that the combination treatment demonstrated bactericidal synergy by disrupting multiple bacterial targets, leading to faster bacterial clearance and preventing regrowth. The enhanced sensitivity of *S. aureus* to MOAL, PIED, MG, and their combinations may be attributed to its thinner peptidoglycan layer and the absence of an outer membrane, facilitating phytochemical penetration. In contrast, *E. coli* and *S. typhi* possess lipopolysaccharide-rich outer membranes that act as permeability barriers, reducing compound efficacy.

The reduction in NO production in LPS-activated macrophages demonstrates the anti-inflammatory potential of all treatment methods. The inhibition rates from combination therapies were significantly higher than those from single extracts. The mixture of MOAL, PIED, and MG at predetermined ratios reduced NO production by 75.1%, outperforming the efficacy of each component tested individually. The combined action of phytochemicals demonstrates a synergistic interaction that enhances the inhibition of NO synthesis beyond additive effects. Experimental results show that the combination of natural agents produces stronger anti-inflammatory effects without cytotoxicity, as validated through parallel viability tests. The findings indicate that developing multi-component plant-based therapies targeting NO-mediated inflammatory pathways may be an effective strategy.

The integrated SwissADME, toxicity, and bioavailability analysis indicates that most MOAL and PIED phytochemicals exhibit favorable physicochemical and safety profiles for oral drug development. Scopoletin, resorcinol, 5-hydroxymethylfurfural, guaiacol, and benzyl alcohol showed an optimal balance of polarity (TPSA ≈ 20–60 Å^2^), low flexibility, high GI absorption, and clean toxicity predictions, making them strong candidates for lead optimization, SAR studies, and in vitro/in vivo validation. In contrast, long-chain lipid derivatives, while displaying high GI absorption and good bioavailability scores, face limitations from excessive molecular flexibility and potential solubility issues, making them less attractive as primary oral leads. D-allose’s poor predicted permeability and furfural’s Ames-positive result further reduce their development priority unless addressed through targeted formulation or supported by strong experimental safety data. Overall, these computational insights provide a clear basis for prioritizing structurally diverse, orally tractable phytochemicals for molecular docking, biological assays, and pharmacokinetic evaluation.

The docking analysis supports the traditional use of MOAL and PIED in treating inflammation, infections, and oxidative stress. Among the tested compounds, Scopoletin emerged as a promising multi-target agent, showing strong affinity for COX-2, TNF-α, and Keap1. These interactions suggest a dual anti-inflammatory and antioxidant mechanism, possibly involving prostaglandin inhibition, cytokine suppression, and Nrf2 activation. Guaiacol and Resorcinol also demonstrated notable binding to DNA gyrase and COX-2, respectively, highlighting their potential roles in antibacterial and anti-inflammatory therapy. Furfural and Furaneol showed moderate but consistent binding to MurA, supporting their involvement in bacterial cell wall inhibition. Although Benzyl alcohol exhibited weak target interactions, its formulation role may still warrant investigation. Overall, these in silico findings provide a strong rationale for further validation through enzymatic assays, cellular studies, and pharmacokinetic profiling to assess their therapeutic relevance.

Overall, the findings highlight the synergistic multi-target therapeutic potential of the MOAL-PIED-MG system. The extraction process efficiently yielded a range of phytochemicals that work together to provide antioxidant, antibacterial, and anti-inflammatory effects exceeding the sum of their actions. The synergistic interactions observed—whether in free radical neutralization, bacterial growth suppression, or inflammatory mediator reduction—suggest that these natural products employ a polypharmacological approach. This approach is highly beneficial in complex pathological conditions. For instance, in wound infections, the MOAL + PIED combination can simultaneously eliminate pathogenic bacteria, neutralize harmful free radicals, and modulate local inflammation, thereby addressing both the cause and consequences of the infection. This multifaceted efficacy is difficult to achieve with a single synthetic drug. Mechanistically, the synergy likely arises from compounds targeting different pathways that collectively improve outcomes. For example, one set of molecules may directly kill bacteria, while another set, such as MG, inhibits bacterial virulence factors by attenuating quorum sensing and virulence in *P. aeruginosa* [96]. Compounds in the extracts could enhance immune response or stress resistance in the host. Additionally, antioxidants in the mixture protect host tissues from oxidative damage during infection, creating a more favorable environment for recovery. These interconnected benefits underscore the therapeutic implications of our findings.

Every research study has its limitations, and this work is no exception; however, these should be seen as opportunities for progress rather than constraints. Our in vitro and in silico findings provide a solid foundation for understanding the pharmacological potential of MOAL, PIED, and MG. SwissADME and molecular docking analyses efficiently identified lead compounds with strong oral drug-likeness, clean safety profiles, and multi-target activity. While in vivo pharmacokinetic and toxicological studies will be needed to confirm these effects, this work offers a clear roadmap for future validation. Variations in phytochemical composition present opportunities for standardization and optimization, ensuring consistency and potency. Most importantly, the synergistic antioxidant, antibacterial, and anti-inflammatory effects demonstrated here underscore the translational promise of these combinations. Together, these results position MOAL–PIED–MG as strong candidates for development into standardized, clinically relevant phytotherapeutics.

## 5. Conclusions

This study shows that extracts from MOAL and PIED exhibit diverse, potent biological effects due to their rich phytochemical composition, either used alone or combined with MG. GC–MS analysis revealed that numerous bioactive compounds, such as phenolics, furans, rare sugars, fatty acids, and terpenoids, contribute significant antioxidant, antimicrobial, and anti-inflammatory properties. The combined formulation of MOAL: PIED: MG at a 1:1:0.1 ratio outperformed individual treatments in radical scavenging, bacterial inhibition, and NO suppression tests while maintaining nontoxic properties. Moreover, molecular docking analysis identified Scopoletin as a key multi-target compound with strong binding affinities to COX-2, TNF-α, and Keap1, indicating potential anti-inflammatory and antioxidant mechanisms. Other constituents, such as Guaiacol, Resorcinol, Furfural, and Furaneol, also demonstrated relevant interactions with microbial and immune-related targets, supporting the extracts’ observed bioactivities. The result showed that both extracts offer therapeutic potential individually and demonstrate enhanced effects when combined in a synergistic phytochemical approach. These multifunctional plant-based therapeutic candidates show broad-spectrum antimicrobial effects against both Gram-positive and Gram-negative bacteria while also demonstrating strong antioxidant and immunomodulatory properties to fight oxidative stress, inflammation, and resistant infections. Future in vivo and clinical research is needed to explore the pharmacological applications and mechanisms of action.

## Figures and Tables

**Table 1:** Combination Ratios of MOAL Extract, PIDE Extract, and MG in Combination Treatments.

Sr. No.	Combination Ratio	Title
1	1:1:0.1	C 1
2	0.5:1.5:0.1	C 2
3	1.5:0.5:0.1	C 3
4	2:0:0.1	C 4
5	0:2:0.1	C 5

Abbreviations: MG, Methyl Gallate; MOAL, *Morus alba;* PIDE, *Pinus densiflora*. The combination ratios of MOAL extract, PIDE extract, and MG were prepared based on weight-to-weight (*w*/*w*) proportions of dried powders.

**Table 2:** Time-dependent extract yield (mg/mL) of MOAL and PIED during a 4 h extraction period with 50% ethanol at a 1:10 (*w*/*v*) solid-to-solvent ratio.

Hours	0	1	2	3	4
Morus (mg/mL)	200	300	400	400	500
Pinus (mg/mL)	100	300	300	400	450

Abbreviations: MOAL, *Morus alba*; PIED, *Pinus densiflora*.

**Table 3:** Phytoconstituents of ethanol-aqueous extract of *Morus alba *(MOAL).

	Compounds	Retention Time	Area (%)	Functions	Formula (Pub Cham)	References
**1**	Acetic acid	5.441	2.69	Antibacterial, Antifungal	C_2_H_4_O_2_/ CH_3_COOH	[37,38,39]
**2**	1-Hydroxy-2-propanone	6.360	1.14	Antibacterial activity, Flavoring agent	C_3_H_6_O_2_	[39,40]
**3**	Methyl-Pyrazine	9.686	0.36	Food additives and Inhibitor on the catalyzed reaction	C_5_H_6_N_2_	[41]
**4**	2-Furanmethanol 3-Furanmethanol	10.363	0.90	Wood furfurylation, Kinetic features	C_5_H_6_O_2_	[42]
**5**	4-Cyclopentene-1,3-dione	11.276	0.31	Induces electrical activity in inexcitable crustacean muscle	C_5_H_4_O_2_	[43]
**6**	Protoanemonine	11.276	0.31	Antifungal agent	C_5_H_4_O_2_	[44]
**7**	Butyrolactone	12.155	0.77	Behavioral effects in baboons	C_4_H_6_O_2_	[45]
**8**	4H-Pyran-4-one,2,3-dihydro-3,5-dihydroxy-6-methyl-	14.317	0.97	Antioxidant activity	C_6_H_8_O_4_	[32]
**9**	Furaneol	16.470	0.58	Anti-biofilm agent	C_6_H_8_O_3_	[36]
**10**	5-amino-1H-Imidazole-4-carboxamide	17.109	0.76	Synergistic antineoplastic activity	C_4_H_7_N_4_O	[46]
**11**	Pentanal	18.003	0.08	A potential biomarker for early detection of ventilator-induced lung injury in rats.	C_5_H_10_O or CH_3_(CH_2_)_3_CHO	[47]
**12**	Ethanamine, *N*-ethyl-*N*-nitroso	19.059	0.73	Increase the level of specific serum IgG (use very low quantity because of toxic effects)	C_4_H_10_N_2_O	[48]
**13**	Butanal, dimethylhydrazine	20.843	0.84	Widely used to fabricate self-healing super-hydrophobic surfaces.	C_6_H_14_N_2_	[49]
**14**	3-methyl-2,5-Piperazinedione	21.530	0.82	Antioxidant activity	C_5_H_8_N_2_O_2_	[50]
**15**	1, 4:3, 6-Dianhydro-. Alpha. -d-glucopyranose	21.634	0.76	Antioxidant activity, Antihistamine activity	C_6_H_8_O_4_	[35]
**16**	5-hydroxymethylfurfural	21.780	10.27	Reported to be a promising candidate for therapy of sickle cell disease.	C_6_H_6_O_3_	[31]
**17**	Hydroquinone	23.048	0.38	Dermatology uses (Skin pigment-lightening agent), Antioxidant	C_6_H_6_O_2_ or C_6_H_4_(OH)_2_	[29,30]
**18**	Resorcinol 1,3-Benzenediol	23.310	10.47	Anthelmintic, Antiseptic	C_6_H_6_O_2_	[51,52]
**19**	Phenol, 3,4-dimethoxy-	27.226	0.44	Antioxidant activity	C_8_H_10_O_3_	[53]
**20**	1,6-anhydro-beta-D-Glucopyranose	29.540	13.54	Important industrial material, helps to produce some chemicals	C_6_H_10_O_5_	[34]
**21**	D-Allose	29.540	13.54	Anticancer activity Cryo-protective effects	C_6_H_12_O_6_	[33,54]
**22**	*N*-Methoxymethyl-*N*-methylacetamide	32.076	3.17	Enhances the anesthetic effects	C_5_H_11_NO_2_	[55]
**23**	2,6-dimethyl-Piperazine	32.16	3.33	It is a key intermediate for the synthesis of sparfloxacin, which is an excellent fluoroquinolone-e antimicrobial agent.	C_6_H_14_N_2_	[56]
**24**	(Z)-2-Butenediamide	32.430	0.22	Anticholinesterase activity, Chelator agents	C_4_H_6_N_2_O_2_	[57]
**25**	4-((1E)-3-Hydroxy-1-propenyl)-2-methoxyphenol	34.911	0.46	-Antioxidant, Antimicrobial, Anti-inflammatory	C_10_H_12_O_3_	[58]
**26**	7-hydroxy-2H-1-Benzopyran-2-one 7-Hydroxycoumarin	37.319	2.43	Anti-parasitic activity	C_9_H_6_O_3_	[59]
**27**	Lidocaine	38.188	0.34	Local anesthetic, Neuropathic pain relief properties	C_14_H_22_N_2_O	[60,61]
**28**	*N*-Hexadecanoic acid	39.242	3.45	Anti-inflammatory, Antibacterial	C_16_H_32_O_2_	[61,62]
**29**	Scopoletin	39.638	3.23	Antibacterial activity, Antifungal activity, Anti-inflammatory activity	C_10_H_8_O_4_	[63,64]
**30**	Ibuprofen, octadecyl ester	40.137	0.30	Anti-inflammatory and analgesic activity	C_31_H_54_O_2_	[65]
**31**	Morpholine, TMS derivative	41.110	0.63	Antioxidants, drug manufacturing, herbicides	C_7_H_17_NOSi	[66]
**32**	(Z, Z)-9,12-octadecadienoic acid	42.531	0.77	Antioxidant activity	C_18_H_32_O_2_	[67]
**33**	Linoelaidic acid	42.531	0.77	Anticancer activity, anti-obesity activity, decreased risk of CVD, anti-inflammatory	C_18_H_32_O_2_	[68]
**34**	1,4-Benzenediol, 2,5-bis(1,1-dimethylethyl)-	42.638	0.33	Anti-CDK1 inhibitory	C_14_H_22_O_2_	[69]
**35**	2-Ethylacridine	47.375	0.27	Founded in plants that have antibacterial and anti-tumor properties	C_15_H_13_N	[70,71]
**36**	2-Methyl-5H-dibenz [b, f] azepine	47.466	0.47	Larvicidal and repellent activities, Preventing the production of ammonia.	C_15_H_13_N	[72,73]

**Table 4:** Phytoconstituents of ethanol-aqueous extract of *Pinus*.

	Compounds	Retention Time	Area (%)	Functions	Formula (Pub Cham)	References
**1**	Acetic acid	5.425	0.78	Antibacterial, Antifungal	CH_3_COOH	[74]
**2**	Phenol	14.316	0.21	Antiseptic, Antibacterial, Antifungal	C_6_H_5_OH	[75]
**3**	1,2-Cyclohexanedione	15.033	1.43	Reacts with arginine residues in proteins	C_6_H_8_O_2_	[76]
**4**	Phenol, 2-methoxy-	17.749	0.2	Antioxidant, Antibacterial, Antifungal	C_10_H_12_O_2_	[77]
**5**	1-Guanidinosuccinimide	18.007	0.25	Potential anticancer activity	C_5_H_7_N_3_O_2_	[78]
**6**	4H-Pyran-4-one, 2,3-dihydro-3,5-dihydroxy-6-methyl-	19.516	0.69	Antioxidant	C_6_H_8_O_4_	[79]
**7**	Hydroquinone	24.144	0.11	Antioxidant, Anti-inflammatory	C_6_H_6_O_2_	[80]
**8**	Phenol, 2-methoxy-3-(2-propenyl)-	28.07	0.69	Antimicrobial	C_10_H_12_O_2_	[81]
**9**	Benzoic acid, 3-hydroxy-	29.622	1.28	Antimicrobial, Anti-inflammatory, Antioxidant	C_7_H_6_O_3_	[82,83]
**10**	Guaiacol	29.882	0.19	Antioxidant, Antibacterial, Antifungal	C_7_H_8_O_2_	[84]
**11**	Guaiacol, 4-butyl-	32.667	0.66	Antioxidant, Antimicrobial	C_11_H_16_O_2_	[85]

**Table 5:** Combined SwissADME, Toxicity, and GI/Bioavailability Data.

**Compounds No.**	***Morus alba* Compound**	**MW (g/mol) < 500**	**HBA < 10**	**HBD < 5**	**Log P < 5**	**Lipinski violations < 1**	**nRB < 10**	**TPSA < 140**	**PAINS Alerts**	**Ames Toxicity**	**hERG I and II**	**Hepatotoxicity**	**Rat Toxicity (LD50, mol/kg)**	**Human Intestinal Absorption (%)**	**GI Absorption**	**Bioavailability Score**
**1**	Resorcinol	110.11	2	2	0.96	0	0	40.46	0	NAT	NO	NO	2.14	86.856	High	0.55
**2**	Hydroquinone	110.11	2	2	0.92	0	0	40.46	0	NAT	NO	NO	2.008	86.856	High	0.55
**3**	5-hydroxymethylfurfural	126.11	3	1	0.91	0	2	50.44	0	NAT	NO	NO	2.283	95.848	High	0.55
**4**	Furfural	96.08	2	0	1.03	0	1	30.21	0	AT	NO	NO	2.429	100	High	0.55
**5**	4H-pyran-4-one	96.08	2	0	1.31	0	0	30.21	0	NAT	NO	NO	2.368	99.374	High	0.55
**6**	D-allose	180.16	6	5	−0.7	0	1	110.38	0	NAT	NO	NO	1.214	21.51	Low	0.55
**7**	Palmitic acid	256.42	2	1	3.85	1	14	37.3	0	NAT	NO	NO	1.44	92.004	High	0.85
**8**	Scopoletin	192.17	4	1	1.86	0	1	59.67	0	NAT	NO	NO	1.95	95.277	High	0.55
**9**	Furaneol	128.13	3	1	1.52	0	0	46.53	0	NAT	NO	NO	2.045	96.011	High	0.85
**10**	4-methoxy-5H-furan-2-one	114.1	3	0	1.36	0	1	35.53	0	NAT	NO	NO	2.095	100	High	0.85
**Compounds No.**	***Pinus* Compound**	**MW (g/mol) < 500**	**HBA < 10**	**HBD < 5**	**Log P < 5**	**Lipinski violations < 1**	**nRB < 10**	**TPSA < 140**	**PAINS Alerts**	**Ames Toxicity**	**hERG I and II**	**Hepatotoxicity**	**Rat Toxicity (LD50, mol/kg)**	**Human Intestinal Absorption (%)**	**GI Absorption**	**Bioavailability Score**
**1**	Guaiacol	124.14	2	1	1.76	0	1	29.46	0	NAT	NO	NO	2.117	93.374	High	0.55
**2**	Phenol	94.11	1	1	1.24	0	0	20.23	0	NAT	NO	NO	2.008	93.055	High	0.55
**3**	Hydroquinone	110.11	2	2	0.92	0	0	40.46	0	NAT	NO	NO	2.153	86.856	High	0.55
**4**	Acetic acid	60.05	2	1	0.63	0	0	37.3	0	NAT	NO	NO	1.774	95.463	High	0.85
**5**	Palmitic acid	256.42	2	1	3.85	1	14	37.3	0	NAT	NO	NO	1.44	92.004	High	0.85
**6**	methyl linoleate	294.47	2	0	3.85	1	15	26.3	0	NAT	NO	NO	1.617	92.66	High	0.85
**7**	Phytol	296.53	1	1	4.85	1	13	20.23	0	NAT	NO	NO	1.848	90.643	High	0.55
**8**	Benzyl alcohol	108.14	1	1	1.66	0	1	20.23	0	NAT	NO	NO	1.994	89.834	High	0.55
**9**	n-hexadecane	226.44	0	0	4.67	1	13	0	0	NAT	NO	NO	1.521	91.046	High	0.55
**10**	Siloxanes	102.21	1	0	1.9	0	0	9.23	0	NAT	NO	NO	2.14	100	High	0.55

MW, Molecular weight; HBA, Hydrogen bond acceptor; HBD, Hydrogen bond donor; Log P, Lipophilicity; nRB, Number of rotatable bonds; TPSA, Topological polar surface area; PAINS, Pan-Assay Interference Compounds; NAT, Non-Ames toxic; AT, Ames toxic.

**Table 6:** Molecular Docking Scores of Phytochemicals with Target Proteins.

Target Protein	PDB ID	Ligand	Binding Score (kcal/mol)
COX-2	5F19	Scopoletin	−5.889
		Resorcinol	−5.840
		Guaiacol	−5.429
TNF-α	2AZ5	Scopoletin	−5.969
		Furaneol	−4.689
MurA	1UAE	Furfural	−5.266
		Furaneol	−5.098
		Benzyl alcohol	−4.721
DNA Gyrase	5MMN	Guaiacol	−5.171
		Scopoletin	−4.798

## Data Availability

All data generated or analyzed during this study are available from the corresponding author upon reasonable request.
